# Anterior chamber depth and axial length affect clinical performance of Spot Vision Screener

**DOI:** 10.5935/0004-2749.20200009

**Published:** 2020

**Authors:** Konuralp Yakar

**Affiliations:** 1 Department of Ophthalmology, Ataturk State Hospital, Sinop, Turkey

**Keywords:** Anterior chamber, Axial length, Amblyopia, Vision di sorders, Vision screening, Retinoscopy, Câmara anterior, Comprimento axial do olho, Ambliopia, Transtornos da visão, Seleção visual, Retinoscopia, Pré-escolar

## Abstract

**Purpose:**

The aim of this study was to evaluate the effect of anterior chamber depth and axial length on clinical performance of the Spot Vision Screener in detecting amblyopia risk factors in children aged 3-10 years.

**Methods:**

A total of 300 eyes from 150 patients aged 3-10 years were prospectively tested with Spot Vision Screener (firmware version 3.0.02.32, software version 3.0.04.06) and a standard autorefractometer (Nidek ARK-1). The anterior chamber depth and axial length were measured with an optical biometer (Nidek AL-Scan). The sensitivity and specificity values for detecting significant refractive errors using the referral criteria of the American Association for Pediatric Ophthalmology and Strabismus were determined. Pearson’s correlation analysis was employed to evaluate the relationship between the Spot Vision results and the anterior chamber depth and axial length.

**Results:**

Compared with the standard autorefractometer results, the Spot Vision Screener’s sensitivity and specificity was 59% and 94%, respectively. The differences between the cycloplegic autorefractometer and the Spot Vision Screener spherical equivalents were negatively correlated with anterior chamber depth (r=-0.48; p<0.001) and axial length (r=-0.45; p<0.001).

**Conclusion:**

The Spot Vision Screener has moderate sensitivity and high specificity, using the criteria of the American Association for Pediatric Ophthalmology and Strabismus. The anterior chamber depth and axial length affect the Spot Vision results.

## Abstract (pt)

**Objetivo:**

O objetivo deste estudo foi avaliar o efeito da profundidade da câmara anterior e do comprimento axial sobre o desempenho clínico do Spot Vision Screener, na deteção de fatores de risco para a ambliopia em crianças de 3 a 10 anos de idade.

**Métodos:**

Um total de 300 olhos de 150 pacientes de 3-10 anos de idade foram prospectivamente testados com o Spot Vision Screener (firmware: 3.0.02.32, software: 3.0.04.06) e com autorefratómetro padrão (Nidek ARK-1). Todas as medições de profundidade e comprimento axial da câmara anterior dos pacientes foram realizadas através de Nidek AL Scan. A sensibilidade e especificidade para a deteção de erros refrativos significativos foram determinadas de acordo com os critérios de referência da Associação Americana de Oftalmologia e Estrabismo Pediátricos. A análise da Correlação de Pearson foi utilizada para avaliar a correlação entre os resultados do Spot Vision e a profundidade ou comprimento axial da câmara anterior dos pacientes.

**Resultados:**

Em comparação com os resultados do autorefratómetro padrão, a sensibilidade do Spot foi de 59% e a especificidade de 94%. As diferenças entre os equivalentes esféricos do autorefratómetro cicloplégico e o Spot Vision Screener foram correlacionados negativamente com a profundidade (r=-0,48; p<0,001) e o comprimento axial (r=-0,45; p<0,001) da câmara anterior dos casos.

**Conclusão:**

O Spot Vision Screener possui uma sensibilidade moderada e uma especificidade elevada utilizando os critérios da Associação Americana de Oftalmologia Pediátrica e Estrabismo; a profundidade da câmara anterior e o comprimento axial dos pacientes afetam os resultados do Spot Vision.

## INTRODUCTION

Amblyopia is the most common cause of unilateral or bilateral vision loss in children, affecting 1.5%-3.6% of the population, and can be treated if diagnosed early. Amblyopia can be classified as strabismic, refractive (anisometropic or isometropic), deprivational, idiopathic, or mixed^(1^,^2)^.

Cycloplegic retinoscopy is commonly emplo yed in cli nics to detect refractive errors and prevent refractive amblyopia in children; however, the technique has its disadvantages, including operator dependency and the need for extensive training^(3^,^4)^. In 2012, the American Academy of Pediatrics, the American Association for Pediatric Ophthalmology and Strabismus (AAPOS), and the American Association of Certified Orthoptists (AACO) recommended early instrument-based pediatric vision screening^(5)^. In 2013, the AAPOS published new guidelines on screening for amblyopia risk factors (ARFs)^(6)^.

Photorefractometers have been shown to be effective for screening refractive errors in preschool children too young to cooperate with fixed autorefractometers^(7^-^9)^.

A photorefractometer is a device that measures the refractive error in both eyes simultaneously by analyzing the patient’s red reflex image with an infrared camera^(10^,^11)^. The device’s sensitivity and specificity for ARF screening have been extensively investigated since their introduction^(12)^.

Previous studies have reported various specificities and sensitivities for several photoscreeners; however, studies have not investigated the factors that affect device performance. The aim of this study was to evaluate the clinical performance of the Spot Vision Screener pediatric photorefractometer (Welch Allyn, Skaneateles Falls, NY, US; firmware version 3.0.02.32, software version 3.0.04.06), a handheld, touchscreen, rechargeable, portable device, for detecting ARFs in Turkish children aged 3 to 10 years (based on the 2013 AAPOS guidelines) and whether anterior chamber depth and axial length could affect the device’s performance.

## METHODS

The study included 300 eyes from 150 patients aged 3-10 years who were admitted to ophthalmology department of Ataturk State Hospital (Sinop, Turkey) for routine eye examination. This prospective study was performed in accordance with the Declaration of Helsinki and was approved by the ethics committee of Ondokuz Mayis University, Samsun, Turkey. A written informed consent was obtained from the parents of all patients after informing them of the study. Patients with congenital cataracts, nystagmus, a history of intraocular surgery, premature retinopathy, or medium opacity, and those who would not cooperate with the devices were excluded.

All patients underwent initial measurements with a Nidek ARK-1 (Tokyo, Japan) fixed autorefracto meter and subsequent measurements with the Spot Vision Screener. The anterior chamber depth and axial lengths of both eyes in all patients were measured using a Nidek AL Scan (Tokyo, Japan) optic biometry device. After taking these measurements, cycloplegia was performed on all patients. During the cycloplegic examination, a drop of 1% cyclopentolate was applied 3 times, in 5-min intervals (at 0, 5, and 10 min); after a 45-min waiting period, and then the measurements were repeated with the autorefractometer. All patients also underwent a complete ophthalmologic and orthoptic evaluation. A single technician performed the measurements, and a single doctor performed all the examinations.

The examinations were conducted in a dimmed room, with the doctor holding the Spot Vision Screener approximately 1 m (3 feet) from the patient. Then, the doctor selected the patient’s age group (6-12 months, 12-36 months, 3-6 years, or 6-20 years) on the device’s home screen. The device then flashed blue and red lights on the screen facing the patient and played a warbling sound to attract the patient’s attention. If the patient pre sented strabismus or the refraction values were not within the reference range specified by the manufacturer, the device alerted the doctor.

The spherical values, astigmatism, and spherical equivalents obtained by cycloplegic refractions of both eyes using the fixed autorefractometer and the noncycloplegic Spot Vision Screener in the patient population were compared. Given that a screening method or device should be noninvasive, the Spot Vision Screener was em ployed as noncycloplegic.The Statistical Package for Social Sciences for Windows, version 15.0 (SPSS Inc., Chicago, IL, US) was used to perform all statistical analyses. The continuous data are presented as means ± standard deviations or median values according to the results of the normality tests. The categorical data are presen ted as numbers and percentages. A Kolmogorov-Smirnov test was performed to assess the data distribution. A Pearson’s or Spearman’s correlation analysis was used to evaluate the correlations between the continuous pa rameters, as appropriate. We employed the 2013 AAPOS Guidelines for detecting ARFs (Table 1) but did not use the reference values of the Spot Vision Screener, in order to compare the current findings of this study with previous reports. Based on the mean refractive error according to the AAPOS ARF criteria, the specificity and sensitivity values of the Spot Vision Screener were calculated.

**Table 1:** Amblyopia risk factors: 2013 American Association for Pediatric Ophthalmology and Strabismus guidelines

	Refractive amblyopia risk factors			
**Age, months**	**Astigmatism**	**Hyperopia**	**Anisometropia**	**Myopia**
12-30	>2.0 D	>4.5 D	>2.5 D	>-3.5 D
31-48	>2.0 D	>4.0 D	>2.0 D	>-3.0 D
>48	>1.5 D	>3.5 D	>1.5 D	>-1.5 D

Nonrefractive mblyopia risk factors^b^.

All ages Manifest strabismus >8PD in primary position.

Media opacity >1 mm.

D= diopters; PD= prism diopter;

a = Additional reporting of sensitivity to detect greater-magnitude refractive errors is encouraged;

b = For all ages.

## RESULTS

Spot Vision Screener, Nidek ARK-1, and Nidek AL Scan measurements were performed for the 300 eyes of the 150 children (71 [47.3%] girls, 79 [52.7%] boys; median age, 7 years [3-10]). The results of the fixed autorefractometer measurements were as follows: median cycloplegic spherical value +1.5 diopter (D) (range, -3.75 to +7.5), median astigmatism of -0.5 D (range, -4.75 to -0.25), and median spherical equivalent of +1.12 D (range, -5.87 to +7.38) (Table 2). Based on the cycloplegic spherical equivalent obtained from a fixed autorefractometer, 19.3% (n=58) of the eyes were myopic, 1.3% (n=4) were ametropic, and 79.4% (n=238) were hyperopic. Therefore, based on the 2013 AAPOS references, we detected ARFs in 23% of the eyes (n=69).

**Table 2:** Median values of refractive parameters using cycloplegic refraction and Spot Vision

	Cycloplegic refraction	Spot vision
Spherical	+1.5 (-3.75 to +7.5)	+0.5 (-3.75 to +6.50)
Astigmatism	-0.5 (-4.75 to -0.25)	-0.75 (-3.00 to -0.25)
Axis	130 (5 to180)	150 (5 to 180)
SE	+1.12 (-5.87 to +7.38)	+0.25 (-5 to +6.25)

SE= spherical equivalent.

The noncycloplegic measurements with the Spot Vision Screener revealed the following: a median spherical value of +0.5 D (range, -3.75 to +6.50), astigmatism of -0.75 D (range -3.00 to -0.25), and median spherical equivalent of +0.25 D (range, -5 to +6.25) (Table 2). Given these values, we detected ARFs in 18% (n=54) of the patients. The Spot Vision Screener had a 59% sensitivity and 94% specificity for noncycloplegic measurements.

The patients had a mean anterior chamber depth of 3.62 ± 0.28 (2.78-4.39) mm and a mean axial length of 22.6 ± 0.93 (19.54-25.00) mm measured by optic biometry.

**Figure 1:**
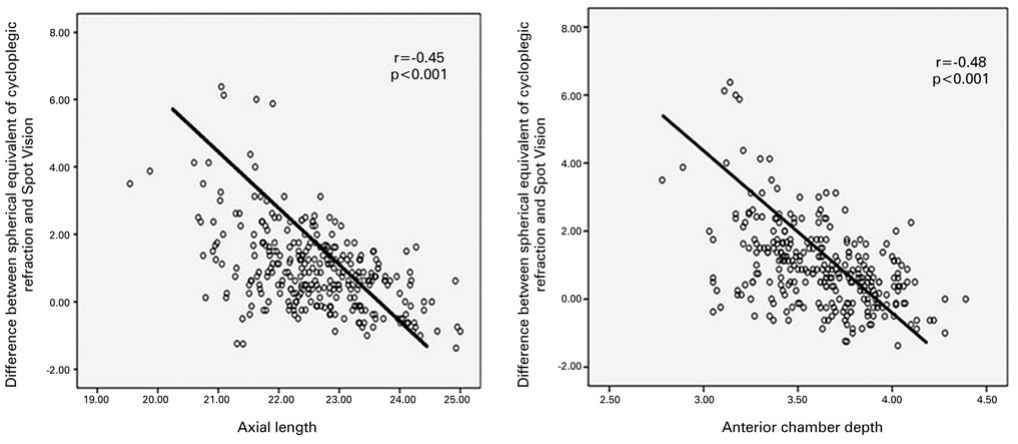
Correlations of axial length and anterior chamber depth with the difference between spherical equivalent of cycloplegic refraction and Spot Vision Screener.

Axial length was negatively correlated with the differences between the spherical equivalent of cycloplegic refraction and the Spot Vision Screener measurements (r=-0.45, p<0.001). The anterior chamber depth was also negatively correlated with the differences between the spherical equivalent of cycloplegic refraction and the Spot Vision Screener measurements (r=-0.48, p<0.001) (Figure 1).

## DISCUSSION

The primary goal of vision screening is to discover individuals at risk and reduce the disease severity as early as possible in childhood^(13)^. Modest et al. demonstrated significant improvement in complete vision screening for children 3-5 years of age with instrument-based vision screening compared with chart-based screening^(14)^. Instrument-based vision screening has also been suggested by AAPOS and AACO for detecting ARFs in early childhood^(15)^.

Numerous studies have found that infrared photorefractors are effective in detecting refraction errors and preventing refractive amblyopia in preschool children^(15^-^20)^. With the development and increasingly widespread use of new pediatric vision screening devices, there is an increasing need to analyze their validity compared with that of existing technology.

The Spot Vision Screener had 59% sensitivity and 94% specificity in the noncycloplegic measurements in our 3-10-year age group compared with a fixed cycloplegic autorefractometer. Forcina et al., in their study comparing ophthalmological examinations and the Spot Vision Screener (software version 2.0.16) for patients aged 6 months to 3 years, reported 89.8% sensitivity and 70.4% specificity^(21)^. Peterseim et al. compared ophthalmological examinations and the Spot Vision Screener (software version 2.0.16) for patients aged 11-221 months and found a sensitivity of 84.8% and a specificity of 70.9%^(11)^. The lower sensitivity and higher specificity observed for the noncycloplegic Spot Vision Screener in the present study could be due to a version change and/or the age difference between the study groups. Paff et al., in their study of noncycloplegic hypermetropia screening, found a sensitivity of 33% and 31% for the Plusoptix S08 photoscreener and Retinomax K-plus 2 autorefractor, respectively^(22)^.

Given that this study population can cooperate with a standard autorefractometer and that retinoscopy is examiner-dependent, I chose cycloplegic autorefraction for this study as the gold standard method for diagnosing ARFs. The Spot Vision Screener was employed as noncycloplegic in the current study because photoscreeners were designed for community screening, and, therefore, most of them are employed as noncycloplegic in daily clinical practice. Previous studies^(11^-^22)^ have also compared cycloplegic retinoscopy with noncycloplegic photoscreeners (Plusoptix S08, Retinomax K-plus, Spot Vision).

In the current study, the differences in the measurements between the spherical equivalents obtained by the cycloplegic autorefractometer and the noncycloplegic Spot Vision Screener were affected by the anterior chamber depth and axial length and were negatively correlated. Based on these results, as the anterior chamber depth and axial length increase, the difference between the spherical equivalents measured with the fixed autorefractometer and the Spot Vision Screener decreases. In other words, measurements by the two devices become closer in individuals with higher anterior chamber depth and axial length values. In cases such as myopia, with a deep anterior chamber and/or long axial length, the Spot Vision Screener appears to be more reliable. Thus, examiners should be on the alert for hyperopic results.

Photorefractors use an infrared camera that analyzes images of the red reflex of an individual’s undilated pupil by assessing the correct alignment of both eyes and estimating the eye’s refractive status. In myopic cases with deep anterior chambers and long axial lengths, the Spot Vision Screener has been more successful in analyzing the red reflex and refractive status. Underestimation of hyperopia (shallow anterior chamber, short axial length) is still a problem with existing devices and could be a bias inherent in current photoscreener technology.

The study has a number of limitations. First, the sample size was relatively small; the results of this study should therefore be validated by larger studies. Second, using the Spot Vision Screener with cycloplegia would have provided more accurate information on the influence of the anatomical factors on the photoscreeners’ performance.

Previous studies have compared photoscreener results with each other and with retinoscopy; however, there is no information on the factors that affect device performance. This study suggests that the patients’ anatomical factors could contribute to device reliability. Physicians should therefore consider anatomical factors when using photoscreeners.
